# CGRP monoclonal antibodies in migraine: an efficacy and tolerability comparison with standard prophylactic drugs

**DOI:** 10.1186/s10194-021-01335-2

**Published:** 2021-10-25

**Authors:** Fenne Vandervorst, Laura Van Deun, Annelies Van Dycke, Koen Paemeleire, Uwe Reuter, Jean Schoenen, Jan Versijpt

**Affiliations:** 1 Department of Neurology, Vrije Universiteit Brussel (VUB), Universitair Ziekenhuis Brussel (UZ Brussel), Brussels, Belgium; 2 Department of Neurology, General Hospital Sint-Jan Bruges, Bruges, Belgium; 3 Department of Neurology, Ghent University Hospital, Ghent, Belgium; 4 Department of Neurology, Charité Universitätsmedizin Berlin, Berlin, Germany; 5 Headache Research Unit, Dept of Neurology-Citadelle Hospital, University of Liège, Liège, Belgium

**Keywords:** Migraine, Episodic migraine, Chronic migraine, Calcitonin gene-related peptide, Monoclonal antibody, Preventive treatment

## Abstract

**Background:**

Several drugs are available for the preventive treatment of both episodic and chronic migraine. The choice of which therapy to initiate first, second, or third is not straightforward and is based on multiple factors, including general efficacy, tolerability, potential for serious adverse events, comorbid conditions, and costs. Recently, a new class of migraine preventive drugs was introduced, i.e. monoclonal antibodies against calcitonin gene-related peptide (CGRP) or its receptor.

**Methods:**

The present article summarizes the evidence gathered with this new migraine preventive drug class from randomized placebo-controlled clinical trials. It further puts this into perspective next to the evidence gained by the most widely used agents for the prevention of episodic and chronic migraine with an emphasis on efficacy and the robustness with which this efficacy signal was obtained.

**Results:**

Although being a relatively new class of migraine preventive drugs, monoclonal antibodies blocking the CGRP pathway have an efficacy which is at least comparable if not higher than those of the currently used preventive drugs. Moreover, the robustness of this efficacy signal is substantiated by several randomized clinical trials each including large numbers of patients. In addition, because of their excellent tolerability and with long-term safety data emerging, they seem to have an unprecedented efficacy over adverse effect profile, clearly resulting in an added value for migraine prevention.

**Conclusions:**

Balancing the data presented in the current manuscript with additional data concerning long term safety on the one hand and cost issues on the other hand, can be of particular use to health policy makers to implement this new drug class in the prevention of migraine.

**Supplementary Information:**

The online version contains supplementary material available at 10.1186/s10194-021-01335-2.

## Background

The treatment of migraine remains an important and challenging task. In a substantial part of patients with episodic migraine (EM), and nearly all patients with chronic migraine (CM), next to an adequate acute medication strategy, a preventive treatment is indicated. Several drugs are currently available for migraine prevention but studies show poor adherence to oral migraine prophylactics [1–4]. Adverse events are cited as the most common reason for discontinuation, next to lack of efficacy. The main causes for their lack of high efficacy and poor tolerability are considered related to the fact that they were not specifically developed for migraine and that most of them have multiple mechanisms of action [5, 6].

The presence of calcitonin gene-related peptide (CGRP) in the trigeminovascular system, the observation of CGRP release during the headache phase of a migraine attack and the induction of a migraine-like headache after intravenous administration of exogenous CGRP, have led to the assumption that CGRP plays a major role in the pathophysiology of migraine [7]. The discovery of this new drug target resulted in the development of the first disease-specific preventive treatment class for both EM and CM, being monoclonal antibodies against the CGRP molecule (eptinezumab, fremanezumab and galcanezumab) or its receptor complex (erenumab) (CGRP mAb).

Over the past few years, several clinical trials with CGRP mAb for the preventive treatment of EM [8–21] and CM [14, 22–27] were performed worldwide. Safety and efficacy results were consistently convincing, resulting in the approval of erenumab, fremanezumab and galcanezumab by both the U.S. Food and Drug Administration (FDA) and the European Medicines Agency (EMA) for the preventive treatment of EM and CM in adults in 2018 and 2019. Eptinezumab received FDA approval in February 2020.

The positioning of these new drugs in the preventive treatment strategy of EM and CM compared to the currently used prophylactic agents remains unclear, since no results from head-to-head trials are yet available. It was already suggested in a recent review that the major added value of the CGRP mAb might be their more favorable efficacy over adverse event profile [28]. The objective of this manuscript is to present a comprehensive overview on currently available results of all four CGRP mAb for the treatment of EM and CM. Secondly, these results are put into perspective next to the available evidence from the most widely used agents for the prevention of EM and CM with an emphasis on efficacy, the robustness of this efficacy signal and tolerability.

## Methods

### Selection of prophylactic migraine drugs

As for the currently used prophylactic agents in EM and CM, only those with a level A evidence as determined by either the European Federation of Neurological Societies (EFNS) [29] or American Academy of Neurology (AAN) [30] were included. Candesartan and amitriptyline were added because they are first line treatments for migraine in several countries. Besides, for both agents a relevant clinical trial was published after the aforementioned guidelines were assembled [31, 32]. Flunarizine was not included in the final analysis despite its level A recommendation in the EFNS guideline. The main reasons are its unavailability in several countries and the fact that the endpoint used in the present manuscript could not be retrieved from any of the performed clinical trials. Timolol also has a level A evidence for the treatment of EM according to the AAN guideline, but not in the EFNS guideline. Since its evidence is considered to be less convincing compared to propranolol and metoprolol [29], and the fact that oral timolol is not available in several European countries, it was not included in the final analysis. As such, the following drugs were selected: propranolol, metoprolol, onabotulinumtoxinA, topiramate, valproate, candesartan and amitriptyline.

### Search methodology

For the currently used prophylactic agents, potential clinical trials were identified by searching the PubMed and Cochrane Library databases (until the 1st of September, 2021). In addition, reference lists of included studies and relevant reviews or meta-analyses were manually screened to identify additional studies that were not found by the computerized search. As for the CGRP mAb, an additional database search on clinicaltrials.gov was performed using the following terms: TEV-48125 or fremanezumab, LY2951742 or galcanezumab, ALD403 or eptinezumab and AMG334 or erenumab. The study search was performed by FVDV, in case of doubt consensus was sought between JV and FVDV.

### Clinical trial selection

Only trials in adults with a randomized (parallel group or cross-over) double-blinded placebo-controlled design studying the efficacy of an agent in monotherapy, of which the full article was available in English, with at least 10 patients in each treatment arm and reporting of the administered dosages were considered. Studies were selected based on title and abstract but deemed suitable for inclusion only after a full-text review.

### Outcome measures

#### Efficacy

The mean reduction of monthly migraine days (MMD) versus placebo was selected as the efficacy endpoint of choice, since it was the primary endpoint in most of the CGRP mAb trials and could be extracted from at least one clinical trial for every other prophylactic agent under study. Moreover, this endpoint was put forward by the International Headache Society (IHS) as one of the primary efficacy measures of choice in drug trials for migraine prophylaxis [33]. If the MMD was calculated at different time points in a trial, only the latest reported was used unless the primary endpoint was set at a different time point.

Results from all 4 CGRP mAb were lumped, considering their comparable mechanism of action and the fact that so far no clear difference in efficacy appears from clinical trials although no head-to-head trials have been performed. Trials with CGRP mAb including patients with difficult to treat migraine were not included in the MMD reduction analysis since no comparable trials are available with the classic prophylactic drugs used for migraine. Since different dosages and dosing regimens were used for every agent, only one dosage was selected per agent for the MMD calculation in order to be consistent. As such, the following dosages were chosen: eptinezumab 300 mg, erenumab 140 mg, fremanezumab 225 mg monthly (with or without loading dose), and galcanezumab 120 mg monthly with a 240 mg loading dose (indicated in bold in Table 1 and 2).

**Table 1:** Results of randomized placebo-controlled clinical trials with CGRP mAb for the treatment of episodic migraine

	Phase	MMD baseline	Exclusion by failed preventives	Study duration (weeks)	Treatment arms	N	MMD change versus placebo	Dropout ratio	Reference	Acronym/NCT
**Eptinezumab**	II	≥5	-	8	placebo	82	-1.0	0%	Dodick et al, 2014	NCT01772524
					1000mg	81		0%		
	III	≥4	-	12	placebo	222	-0.7	3%	Ashina et al, 2020	PROMISE-1
					100mg	221		3%		
					**300mg**	222	-1.1	2%		
**Fremanezumab**	II	≥8	>2	12	placebo	104	-2.8	0%	Bigal et al, 2015	NCT02025556
					**225mg**	96		4%		
					675mg monthly	96	-2.6	2%		
	III	≥4	≥2	12	placebo	294	-1.5	2%	Dodick et al, 2018	HALO
					**225mg**	289		1%		
					675mg	291	-1.3	2%		
	II/III	≥4	≥2	12	placebo	117	-3.0	1%	Sakai et al, 2021	NCT03303092
					**225mg**	121		1%		
					675mg	119	-3.0	0%		
**Galcanezumab**	II	≥4	>2	12	placebo	110	-1.2	1%	Dodick et al, 2014	NCT01625988
					150mg every 2 weeks	107		0%		
	II	≥4	>2	12	placebo	137	-0.9	0%	Sklajarevski et al, 2018	NCT02163993
					120mg	70		0%		
					300mg	67	-0.9	1%		
	III	≥4	>2	24	placebo	433	-1.9	2%	Stauffer et al, 2018	EVOLVE-1
					**120mg (**^a^**240mg)**	213		4%		
					240mg	212	-1.8	3%		
	III	≥4	>2	24	placebo	461	-2.0	2%	Sklajarevski et al, 2018	EVOLVE-2
					**120mg (**^a^**240mg)**	231		2%		
					240mg	223	-1.9	4%		
**Erenumab**	II	≥4	>2	12	placebo	153	-1.1	1%	Sun et al, 2016	NCT01952574
					70mg	106		3%		
	II	≥4	>2	24	placebo	136	-2.3	1%	Sakai et al, 2019	NCT02630459
					70mg	135		1%		
					**140mg**	137	-1.9	0%		
	III	≥4	>2	24	placebo	319	-1.4	3%	Goadsby et al, 2017	STRIVE
					70mg	314		2%		
					**140mg**	319	-1.9	2%		
	III	≥4	>2	12	placebo	289	-1.0	0%	Dodick et al, 2018	ARISE
					70mg	283		2%		
	III	≥4	>2	12	placebo	338		1%	Wang et al, 2021	EMPOwER
					70mg	338	-1.1	0%		
					**140mg**	224	-1.7	0%		

Unless indicated differently, dosing is monthly for erenumab, galcanezumab and fremanezumab 225mg and every 3 months for eptinezumab and fremanezumab 675mg

*MMD* monthly migraine days (for the group average MMD reduction only dosages in bold were used), *NA* not available

^a^loading dosage

**Table 2:** Results of randomized placebo-controlled clinical trials with CGRP mAb for the treatment of chronic migraine

	Phase	Exclusion by unremitting headaches	Exclusion by failed preventives	Study duration (weeks)	Treatment arms	N	MMD change versus placebo	Number of dropouts	Dropout ratio	Reference	Acronym/NCT
**Eptinezumab**	II	-	-	12	placebo	121	-2.1	0	0%	Dodick et al, 2019	NCT02275117
					100mg	123		2	2%		
					**300mg**	120	-2.6	4	3%		
	III	+	-	12	placebo	366	-2.0	2	1%	Lipton et al, 2020	PROMISE-2
					100mg	356		3	1%		
					**300mg**	350	-2.6	8	2%		
**Fremanezumab**	II	-	≥3	12	placebo	89	-1.7	1	1%	Bigal et al, 2015	NCT02021773
					**225mg (**^a^**675mg)**	88		4	5%		
					900mg monthly	86	-2.0	3	3%		
	III	+	≥2	12	placebo	375	-1.7	8	2%	Silberstein et al, 2017	HALO
					675mg	376		5	1%		
					**225mg (**^a^**675mg)**	379	-1.8	7	2%		
	II/III	+	≥2	12	placebo	191	-1.3	3	2%	Sakai et al, 2021	NCT03303079
					675mg	191		1	1%		
					**225mg**	189	-2.1	0	0%		
**Galcanezumab**	III	+	>3	12	placebo	558	-2.1	6	1%	Detke et al, 2018	REGAIN
					**120mg (**^a^**240mg)**	278		3	1%		
					240mg	277	-1.9	2	1%		
**Erenumab**	II	+	>3	12	placebo	282	-2.4	2	1%	Tepper et al, 2017	NCT02066415
					70mg	190		0	0%		
					**140mg**	188	-2.4	2	1%		

Unless indicated differently, dosing is monthly for erenumab, galcanezumab and fremanezumab 225mg and every 3 months for eptinezumab and fremanezumab 675mg

*MMD* monthly migraine days (for the group average MMD reduction only dosages in bold were used)

^a^loading dosage

For the standard prophylactics, if efficacy results from different dosages were available, only those from dosages commonly used in clinical practice were included. As such, efficacy results from the following daily dosages were used for the MMD calculation: propranolol 80-160 mg, metoprolol 100-200 mg, topiramate 100 mg for EM and 50-200 mg for CM, valproate 250-1500 mg, candesartan 8-16 mg, amitriptyline 25 mg and onabotulinumtoxinA at the injection sites and dosing according to the PREEMPT trials [34]. Since there is not enough evidence that propranolol and metoprolol have a different efficacy profile, both agents were lumped in the final analysis (further referred to as beta-blockers).

The reduction in MMD versus placebo for every agent was calculated by averaging and weighing the MMD reduction according to the total number of patients in the respective trials (so larger trials contributed more to the final average MMD reduction). Only patients who entered the placebo-controlled phase of the study were counted, as such excluding screen failures during the screening or baseline phase.

#### Tolerability

For each clinical trial, dropout rates for both the active treatment and placebo arm were calculated, if available. Dropout rates were calculated as follows: the number of patients who discontinued the clinical trial due to side-effects between randomization and the time the primary endpoint was reached, divided by the total number of subjects who were randomized in the same treatment arm. The dropout rate for every prophylactic agent was calculated by summing all dropout rates across every trial (so larger trials contributed more to the final dropout rate).

### Data extraction

The respective data extraction was independently performed by at least two authors (LVD, AVD and FV for the standard prophylactic drugs and JV and FV for the CGRP mAb). Any unclarities or disagreements were resolved by consensus between FV and JV.

## Results

### Monoclonal antibodies targeting the CGRP pathway

The first phase 2 trials with CGRP mAb were published in 2014 (eptinezumab and galcanezumab) [8, 16]. To date, results from six phase 2 and eight phase 3 trials are available for EM [8–21] next to three phase 2 and four phase 3 trials for CM [14, 22–27] (*Table* *1**and* *2*). The average reductions in MMD for CGRP mAb versus placebo in EM and CM, were respectively 1,9 and 2,2 days. None of the trials performed did not reach its primary endpoint, moreover each trial included a large number of patients.

In addition, the efficacy of CGRP mAb was specifically studied in patients with difficult-to-treat migraine, implicating a failure in terms of efficacy and/or tolerability of two to four preventive treatments, in four randomized placebo-controlled trials [35–38] (*Table* *3*). In three out of four studies patients with both EM and CM were included [35, 36, 38]. Worth noting is the fact that in two out of three already completed trials, the MMD reduction versus placebo was relatively high. The remarkably low placebo response in these trials could have contributed to this [35–37].

**Table 3:** Results of randomized placebo-controlled clinical trials with CGRP mAb for the treatment of difficult-to-treat migraine

	MHD	Exclusion by unremitting headaches	Study duration (weeks)	Failed preventives required	Treatment arms	N	MMD change versus placebo	Dropout ratio	Reference	Acronym
**Fremanezumab**	6-30	+	12	2-4	placebo	279	-3.1	1%	Ferrari et al, 2019	FOCUS
					675mg	276		0%		
					225mg^a^	283	-3.5	1%		
**Galcanezumab**	4-30	+	12	2-4	placebo	230	-3.1	0%	Mulleners et al, 2020	CONQUER
					120mg (^b^240mg)	232		0%		
**Erenumab**	4-14	-	12	2-4	placebo	125	-1.6	0%	Reuter et al, 2018	LIBERTY
					140mg	121		0%		
**Eptinezumab**	4-30	-		2-4	placebo	280	Recruiting			DELIVER
					100mg	280				
					300mg	280				

Unless indicated differently, dosing is monthly for erenumab, galcanezumab and fremanezumab 225mg and every 3 months for eptinezumab and fremanezumab 675mg

*NA* data not available, *MHD* monthly headache days, *MMD* monthly migraine days

^a^the chronic migraine subgroup received 675mg at the first month

^b^loading dosage

### Currently used prophylactic agents

An overview of all included clinical trials for every prophylactic agent and their core results are presented in the *Supplementary material**.* Below we give an overview of every prophylactic agent studied and highlight particular findings.

#### Candesartan

Two trials compared candesartan 8-16 mg with placebo for the treatment of migraine. The first [39] was published in 2003 and included 60 EM patients. The second [40] admitted patients with both EM and CM, but no separate subanalysis was made. The weighted average MMD reduction compared to placebo was 0,9 days.

#### Topiramate

Topiramate is a frequently used prophylactic agent in both EM and CM worldwide. Eight placebo-controlled trials for the treatment of EM [41–48] and three for the treatment of CM [49–51] were included. Dosages varied between 50 and 200 mg. One EM trial did not reach its primary efficacy endpoint [47]. In this trial a daily dose of 200 mg was studied. For EM the MMD reduction versus placebo was 1,2 days and for CM 1,8 days. Strikingly, in nearly all trials a relatively high dropout rate (all causes) among topiramate (range 13 to 62%) but even placebo (range 10 to 48%) treated patients was found. Looking only at dropouts due to side-effects the difference was 14% compared to placebo.

#### Valproate

Six placebo-controlled trials including in total 436 EM valproate treated subjects were withheld [52–57]. The MMD reduction compared to placebo was 1,7 days. Dropout rates among patients treated with valproate varied between 3 and 19%, compared to 0 and 9% in the placebo group.

#### Beta-blockers

For propranolol the first trial was conducted in 1972, for metoprolol in 1983. Results from 18 randomized, placebo-controlled trials with propranolol [40, 41, 56, 58–72] and 4 with metoprolol for the preventive treatment of EM were included [73–76]. A total number of 1035 patients were treated with beta-blockers, 886 with propranolol and 149 with metoprolol. The MMD reduction compared to placebo was 0,7 days for propranolol (based on two studies) and 1,6 days for metoprolol (based on two studies), yielding an averaged and weighted MMD reduction of 0,9 days for beta-blockers.

Dropout rates varied between 0 and 20% for propranolol (compared to 0–10% for placebo) and between 0 and 4% for metoprolol (compared to 0–3% for placebo).

#### Amitriptyline

Three randomized placebo-controlled trials were included [32, 77, 78], studying a total number of 308 amitriptyline treated patients (dosage of 25 to 100 mg). In one study both EM and chronic daily headache patients were included [78]. The results of this 20-week trial were considered negative since a significant reduction in headache frequency compared to placebo could only be observed at 8 weeks, but not at 12, 16, or 20 weeks. Overall dropout rates (all causes) were strikingly high for both amitriptyline and placebo, up to 48% for amitriptyline and 54% for placebo. Looking only at dropouts due to side-effects the difference was 5% compared to placebo. In only one trial a MMD change was used as an endpoint, resulting in a MMD reduction of 1,1 days in the amitriptyline treated patients (*n* = 59) compared to placebo.

#### OnabotulinumtoxinA

Two large, randomized controlled trials including 688 patients treated with onabotulinumtoxinA (155-195 U) for the treatment of CM (following a fixed site injection protocol) were published in 2010 being the PREEMPT 1 and 2 trial [79, 80]. The PREEMPT 1 trial did not reach its primary endpoint, the PREEMPT 2 did and the pooled analysis also resulted in significant improvements compared with placebo in multiple headache symptom measures [34]. Dropout rates were 3% in the treatment arm versus 1% in the placebo arm. The MMD reduction compared to placebo was 2,0 days.

### Global overview

**Fig. 1:**
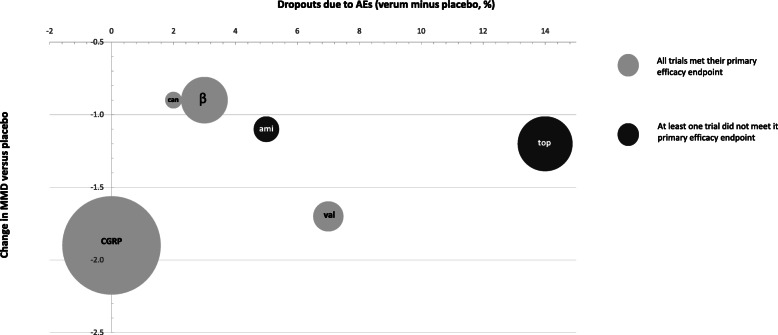
dropouts due to AEs and change in MMD versus placebo in episodic migraine patients. The size of the circle corresponds to the number of patients that were treated with the prophylactic agent across all RCTs. can: candesartan; ami: amitriptyline; top: topiramate: val: valproate; CGRP: CGRP mAb; β: beta-blockers; RCT: randomised controlled trial; MMD: monthly migraine days

**Fig. 2:**
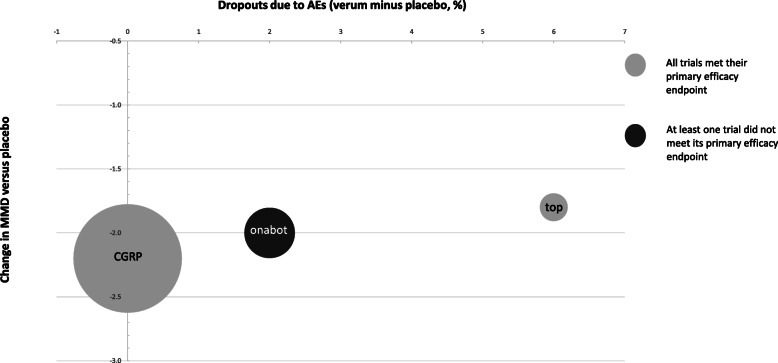
dropouts due to AEs and change in MMD versus placebo in chronic migraine patients. The size of the circle corresponds to the number of patients that were treated with the prophylactic agent across all RCTs. top: topiramate; onabot: onabotulinumtoxinA; CGRP: CGRP MAb; RCT: randomised controlled trial; MMD: monthly migraine days

Figures 1 and 2 illustrate, for EM and CM respectively, per agent the number of patients that were treated in clinical trials, the dropouts due to side-effects compared to placebo and their calculated weighted average MMD reduction compared to placebo.

*Table* *4* gives an overview of the obtained results for both EM and CM.

**Table 4:** overview of trial results for all studied prophylactic agents in both episodic and chronic mgiraine

		MMD reduction (range)	N	RCTs	negative RCTs	dropouts verum, % (range)	dropouts placebo, % (range)	dropouts verum minus placebo, %
**EM**	**CGRP mAb**	1.9 (1.1-3,0)	4632 (1852)	14 (9)	0	2 (0-4)	2 (0-3)	0
	**candesartan**	0.9 (0.6-1.2)	132	2	0	2 (1-2)	0 (0-0)	2
	**topiramate**	1.2 (0.7-1.6)	1433 (577)	8 (4)	1	22 (7-35)	9 (0-12)	14
	**valproate**	1.7 (1.3-2.6)	436 (235)	6 (3)	0	12 (3-19)	5 (0-9)	7
	**beta-blockers**	0.9 (0.6-2.1)	1035 (349)	22 (4)	0	6 (0-20)	3 (0-10)	3
	**amitriptyline**	1.1	308 (59)	3 (1)	1	11 (9-12)	6 (3-7)	5
**CM**	**topiramate**	1.8 (1.5-3.7)	211 (197)	3 (2)	0	12 (7-19)	6 (0-11)	6
	**CGRP mAb**	2.2 (1.7-2.6)	3191 (1592)	7	0	1 (0-5)	1 (0-2)	0
	**onabotulinumtoxinA**	2.0 (1.5-2.4)	688	2	1	3 (2-3)	1 (0-2)	2

*EM* episodic migraine, *CM* chronic migraine, *MMD* relative reduction in migraine days per month versus placebo

N: number of patients treated (receiving verum) in randomized controlled trials (in brackets the total number of patients treated with verum for which the MMD was calculated, only mentioned if the number is smaller)

RCTs: number of randomised controlled trials (in brackets the number of trials for which MMD data were available and used for the group calculation, only mentioned if the number is smaller)

## Discussion

MMD reductions of all assessed prophylactic drugs in EM and CM compared to placebo varied between 0,9 and 2,2 days. When looking at EM and CM separately, the values range between 0,9 and 1,8 days for EM, and 1,9 and 2,2 days for CM. In both EM and CM, the highest MMD reduction was found for the CGRP mAb. The true clinical efficacy of CGRP mAb might even be higher since overall a high placebo response was reached in most of the trials probably related to their more invasive route of administration [81]. However, whether this small difference reflects a clinically meaningful difference remains unclear, since a head-to-head statistical comparison of the studied prophylactic agents is limited by several factors.

First, there is an enormous variation in trial design. As such, the chosen efficacy parameter, MMD reduction, could not be retrieved in all trials of the currently used prophylactic drugs. In mainly the older trials, other efficacy parameters were used (for example migraine attacks). Indeed, MMD was only proposed in 1985 as an alternative efficacy endpoint for the number of migraine attacks [82]. This heterogeneity of outcome measures is a well-known problem among drug trials dealing with migraine prophylaxis, rendering a formal quantitative meta-analysis not feasible.

Second, large differences in both the number of patients treated with the preventive agent and the number of trials performed were seen. The studied sample size ranged for example between 132 patients for candesartan and 3191 (CM) or 4632 (EM) for CGRP mAb. As for the number of trials performed, this ranged between only two (both onabotulinumtoxinA for CM and candesartan) and 22 for beta-blockers. This also limits a formal statistical comparison of prophylactic agents.

Third, a huge variation in methodological quality of included studies of the currently used prophylactic agents has been demonstrated [83–86]. As such, only valproate, metoprolol, propranolol and topiramate have a level A recommendation for the treatment of EM [29, 30]. However, even for those 4 prophylactic agents several possible biases were identified by previous meta-analyses [83–86]. Recent meta-analyses evaluating the efficacy of CGRP mAb on the other hand showed that the trial quality assessment was consistently more homogeneous with an overall low risk of bias [87–91].

Efficacy results from all CGRP mAb were lumped. One should however be aware of the important differences across trials, concerning amongst others the required and maximum number of headache days at baseline, the allowance of medication overuse, the number of previously failed or currently preventive agents allowed, study duration (varying between three and six months), the chosen primary endpoint and the way this was calculated, the definition of a migraine day, all this combined with different dosing schemes.

Among the currently used prophylactics, the highest dropout rates compared to placebo were seen in patients treated with amitriptyline, valproate or topiramate. These high dropout rates seem to be consistent with both clinical practice and with data about migraine prophylaxis adherence, in which a substantial higher rate of discontinuation was seen among patients treated with topiramate and amitriptyline compared to propranolol [4]. One has to be aware however that the overall tolerability and safety story is not fully reflected by ‘dropouts due to side-effects’ in a clinical trial. As for the oral prophylactic drugs, side effects like depressive mood, weight changes or nephrolithiasis might not be completely captured during a clinical trial. Even so, the real-world side effect profile of CGRP mAb is an evolving area of research where for example the development of hypertension or worsening of preexisting hypertension due to erenumab needs to be further elucidated in the near future. Finally, the concurrent high overall dropout rates in the amitriptyline and topiramate trials is remarkable, and might reflect a lower trial quality although no firm conclusions can be drawn.

## Conclusions

CGRP mAb have an efficacy which is at least comparable to the efficacy of the currently used preventive drugs where the robustness of this efficacy signal is substantiated by several randomized clinical trials each containing large numbers of patients. Because of their excellent tolerability and ease of use, the major added value of CGRP mAb, compared to the classical preventive anti-migraine drugs, seems therefore to be their unprecedented high efficacy over adverse effect profile. The high cost of CGRP mAb urges further research both exploring their cost-effectiveness and subgroups of patients who are likely to benefit most. Combining all this information with additional data concerning long term safety, can be of particular use to health policy makers in order to be able to provide guidelines on how to implement this new class of drugs in the prevention of EM and CM.

## Supplementary Information

**Additional file 1: Table S1.** overview of all included trials of currently used prophylactics excluding CGRP-based therapies.

## Data Availability

All data generated or analysed during this study are included in this published article and its supplementary information files.

## Abbreviations

AAN: American Academy of Neurology
EM: Episodic migraine
EMA: European Medicines Agency
CM: Chronic migraine
EFNS: European Federation of Neurological Societies
FDA: Food and Drug Administration
IHS: International Headache Society.
CGRP: Calcitonin gene-related peptide
mAb: monoclonal antibody
MMD: Monthly migraine day
RCT: Randomized controlled trial
